# Variations in atherosclerosis and remodeling patterns in aorta and carotids

**DOI:** 10.1186/1532-429X-12-10

**Published:** 2010-03-05

**Authors:** Katsumi Hayashi, Venkatesh Mani, Ajay Nemade, Silvia Aguiar, John E Postley, Valentin Fuster, Zahi A Fayad

**Affiliations:** 1 Imaging Science Laboratories, Translational and Molecular imaging Institute, Department of Radiology, Mount Sinai School of Med, New York, NY, USA; 2 College of Physicians and Surgeons, Columbia University, New York, NY, USA; 3 Department of Medicine, Mount Sinai School of Medicine, New York, NY, USA

## Abstract

**Background:**

Atherosclerosis is a progressive disease that causes vascular remodeling that can be positive or negative. The evolution of arterial wall thickening and changes in lumen size under current "standard of care" in different arterial beds is unclear. The purpose of this study was to examine arterial remodeling and progression/regression of atherosclerosis in aorta and carotid arteries of individuals at risk for atherosclerosis normalized over a 1-year period.

**Methods:**

In this study, 28 patients underwent at least 2 black-blood in vivo cardiovascular magnetic resonance (CMR) scans of aorta and carotids over a one-year period (Mean 17.8 ± 7.5 months). Clinical risk profiles for atherosclerosis and medications were documented and patients were followed by their referring physicians under current "standard of care" guidelines. Carotid and aortic wall lumen areas were matched across the time-points from cross-sectional images.

**Results:**

The wall area increased by 8.67%, 10.64%, and 13.24% per year (carotid artery, thoracic aorta and abdominal aorta respectively, p < 0.001). The lumen area of the abdominal aorta increased by 4.97% per year (p = 0.002), but the carotid artery and thoracic aorta lumen areas did not change significantly. The use of statin therapy did not change the rate of increase of wall area of carotid artery, thoracic and abdominal aorta, but decreased the rate of change of lumen area of carotid artery (-3.08 ± 11.34 vs. 0.19 ± 12.91 p < 0.05).

**Conclusions:**

Results of this study of multiple vascular beds indicated that different vascular locations exhibited varying progression of atherosclerosis and remodeling as monitored by CMR.

## Introduction

Atherosclerosis is a progressive disease that causes vascular remodeling i.e. changes in the vessel wall of arteries [1]. This remodeling can be positive or negative[2,3]. Atherosclerosis is a major cause of morbidity and mortality world-wide with the most serious outcomes being myocardial infarction, stroke, and death[4,5]. Atherosclerosis affects all vascular beds, including the coronary, carotid, aorta, and peripheral arteries and is present years before a cardiovascular event [6]. The evolution of arterial wall thickening and changes in lumen size i.e., the natural progression/regression of disease under current "standard of care" in different arterial beds are unclear.

Recently, black blood cardiovascular magnetic resonance (CMR) has become a useful tool for non-invasively evaluating vascular wall area and lumen area [7-16]. CMR findings have been extensively validated against pathology in ex vivo studies of carotid, aorta, and coronary artery specimens obtained at autopsy and using experimental models of atherosclerosis [17-19]. Previous studies have examined plaque progression/regression in carotids, aorta or femoral arteries separately but very few studies have examined multiple vascular beds in the same patient population. Furthermore, a majority of previous studies follow specific lesions in the artery of interest over time, but do not evaluate changes in plaque burden over the entire vessel of interest [20,21]. Most previous studies have also examined relatively advanced atherosclerotic lesions while looking for progression/regression and effect of treatment [22,23]. The purpose of this study was to examine arterial remodeling and progression/regression of atherosclerosis in multiple vascular beds (aorta and carotid arteries) of individuals at risk for atherosclerosis over the entire artery of interest and not a specific lesion (carotids, abdominal aorta, thoracic aorta) normalized over a one-year period.

## Methods

### Study population

This study was approved by the local institutional review board. Informed consent was obtained from all subjects prior to participation in the study. Three hundred subjects aged 9 years or older and with at least two risk factors for atherosclerosis were recruited serially from January 2003 - December 2006 into an imaging study. Patients were recruited from the offices of local physicians in the New York area and at the Mount Sinai Medical Center. The local physicians screened patients based on their traditional risk factors for atherosclerosis (age, gender, smoking status, hypertension, diabetes, lipid profile, and carotid intima-media thickness measures (IMT)). Any patient who had at least two risk factors for atherosclerosis (not including older age) was recruited into the imaging study. The following criteria were used to determine a positive risk factor: A mean IMT > 1.1 mm, or a focal structure that encroached into the arterial lumen by at least 0.6 mm represented a positive IMT. A patient was considered to be hypertensive if either systolic or diastolic blood pressure was greater than 140 and 100 mm HG respectively or the patient was on antihypertensive medication. A patient was considered hypercholesterolemic if total cholesterol was > 200 mg/dl, and LDL was > 160 mg/dl or if the patient was on a statin. Fasting plasma glucose levels of more than 126 mg/dl on two or more tests on different days or HbA1c > 6.5 indicated diabetes.

From this sample of 300 patients who underwent one or more CMR scans of both aorta and carotid, 28 subjects who underwent CMR of both aorta (thoracic and abdominal) and carotids (left and right) two or three times over a two year period were included in this retrospective analysis. Study subjects were asked, prior to the first CMR examination, to complete a detailed health questionnaire and physical examination (table 1). Patients needed to have their statin data at the time of imaging available for inclusion in the retrospective analysis. Exclusion criteria included: 1) a repeat CMR within 6 months; 2) age less than 20 years old. All patients were under the supervision of their respective primary care physicians and were following current "standard of care" guidelines.

**Table 1:** Baseline clinical data

Demographics and risk factors	Carotid (n = 23 Mean ± S.D (Range/dosage or % if possible)	Thoracic (n = 19 Mean ± S.D (Range/dosage or % if possible)	Abdominal (n = 15 Mean ± S.D (Range/dosage or % if possible)	p-value
Age (years)	59.4 ± 10.3 (75-27)	59.8 ± 12.0 (85-35)	63.2 ± 12.3 (85-35)	ns
Male sex (%)	66.7	68.4	60.0	ns
Height (m)	1.72 ± 0.08 (1.83-1.55)	1.72 ± 0.10 (1.89-1.55)	1.70 ± 0.09 (1.83-1.55)	ns
Weight (kg)	77.5 ± 16.8 (114-52)	78.5 ± 17.1 (123-52)	79.5 ± 16.0 (114-52)	ns
Body Mass Index (kg/m2)	26.0 ± 4.9 (39.2-18.9)	26.3 ± 4.0 (34.5-18.9)	27.4 ± 5.0 (39.2-18.9)	ns
Hypertension (%)	45.0	38.9	35.7	ns
Diabetes (%)	31.8	26.3	40.0	ns
Smoking status (%)				
Active	4.5	11.1	14.3	ns
Quit	45.0	38.9	28.6	ns
Never	49.5	50.0	57.1	ns
History of CAD (%)	27.3	21.1	26.7	ns
Hypercholesterolemia (%)	35.3	29.4	29.4	ns
Statins use (%)	41.7	42.1	40.0	ns

Statins (type)				
Atorvastatin	80.0 (5-10)	87.5 (5-10)	100.0 (5-40)	ns
Pravastatin	10.0	12.5	0.0	ns
Rosuvastatin	10.0	0.0	0.0	ns
				
Statins (dosage)				
None	60.0	62.5	50.0	ns
Low	40.0	37.5	33.3	ns
High	0.0	0.0	16.7 ns	
				
Total cholesterol (mg/dl)	189.0 ± 52.2 (122-287)	191.3 ± 47.3 (122-287)	186.3 ± 56.2 (122-287)	ns
LDL cholesterol (mg/dl)	115.6 ± 42.2 (62-202)	113.0 ± 41.3 (65-202)	106.0 ± 50.8 (62-202)	ns
HDL cholesterol (mg/dl)	51.6 ± 15.9 (30-86)	51.3 ± 16.8 (30-86)	55.9 ± 17.6 (32-86)	ns
Triglycerides (mg/dl)	110.6 ± 59.7 (46-234)	135.9 ± 86.6 (46-335)	154.4 ± 132.9 (46-500)	ns

CAD: Coronary Artery Disease; low dose: atorvastatin 5-10 mg; high dose: atorvastatin 40 mg. Data are mean ± S.D or % a unpaired t-test

Three separate vascular beds were analyzed for this study (carotid arteries, thoracic aorta and abdominal aorta). Ten subjects had carotid artery images, thoracic aorta images and abdominal aorta images. Five subjects had carotid artery images and thoracic images. One subject had carotid artery images and abdominal aorta images. Three subjects had thoracic and abdominal aortic images, seven subjects had only carotid images, one subject had only thoracic aorta images and one subject had only abdominal aorta images.

The total number of subjects for carotid artery, thoracic aorta and abdominal aorta were 23, 19 and 15 respectively.

The duration between first imaging and second imaging or first imaging and third imaging was 17.1 ± 7.6 months (Range 7.6-48.6) in carotids, 17.1 ± 6.7 months (Range 8.8-30.0) in thoracic aorta and 20.9 ± 7.8 months (Range 9.3-32.0) for abdominal aorta. In the case of more than 2 imaging visits, the last visit (furthest interval apart from Visit 1) was chosen as the 2^nd ^visit for calculating rates of progression/regression.

The percentage of individuals with statin use was 41.7% in carotid artery, 42.1% in thoracic aorta and 40.0% in abdominal aorta. Statin dose was unavailable from >50% of subjects. High dose statin was used only in 1 subject in abdominal aorta.

### CMR protocol

All MR images were obtained on a 1.5T whole body MR imaging system (Siemens Sonata, Erlangen, Germany) that was running Numaris 4.0 operating system. The system had a maximum gradient amplitude of 40 mT/m and a slew rate of 200 mT/m/ms. The integrated body coil was used for transmission. For the carotid images a custom built 4-channel carotid array was used for signal reception [13,24] and for the aorta images, a 6 channel cardiac coil in conjunction with the spine array was used for signal reception.

### Carotid Imaging

Twelve to 24 non-overlapping cross sectional slices centered on the carotid bifurcation were obtained using the rapid extended coverage double inversion recovery turbo spin echo black blood (REX) pulse sequence[25]. Imaging parameters were as follows: proton density weighted (PDW) non-gated sequence imaging 12 slices simultaneously (TR/TE = 2130/5.6 ms), with a field of view of 12 × 12 cm, bandwidth of 488 Hz/pixel, matrix size of 256 × 256, a turbo factor of 15 and 2 signal averages. A chemical shift suppression pulse was used to suppress signal from perivascular fat, not affecting the signal from intraplaque lipids.

The image slice was 3 mm and the gap between image slices was 0.3 mm.

### Aorta Imaging

Twenty five to 30 transverse images from the origin of the left subclavian artery to the level of the diaphragm were obtained. Some subjects got images of the abdominal aorta using the same method extending from the level of the diaphragm to the level of the iliac bifurcation.

The imaging parameters were similar for carotid imaging with the exception of field of view and slice thickness which were 25 cm and 5 mm respectively. The gap between slices was 0.5 mm. The total examination lasted 60 to 90 minutes.

### MR imaging analysis

After MR images were acquired, they were transferred to a dedicated MR workstation for analysis. An experienced reader (KH) analyzed all the images. The experienced observer also qualitatively scored the image quality, using a scale from 1 to 5, with 5 representing best quality. Sections with an CMR quality rating of ≤ 2 were excluded from the analysis [13,19]. The acceptance rate for images based on these criteria was 72.4% for carotids, 75.6% for thoracic aorta and 73.4% for abdominal aorta for all imaging time points. There were similarly no significant differences between image quality scores between the various imaging time points. The outer and inner vessel wall contours were manually traced for the aorta and carotid artery using a custom software program (VesselMass, Leiden University Medical Center, The Netherlands)[26]. Wall area, lumen area, and total vessel area were automatically calculated based on the contours drawn by the software program. The normalized wall index (NWI) was calculated by dividing the wall area by the total vessel area.

Registration of images between the time points was performed manually by an expert observer using various fiducial landmarks.

**Figure 1:**
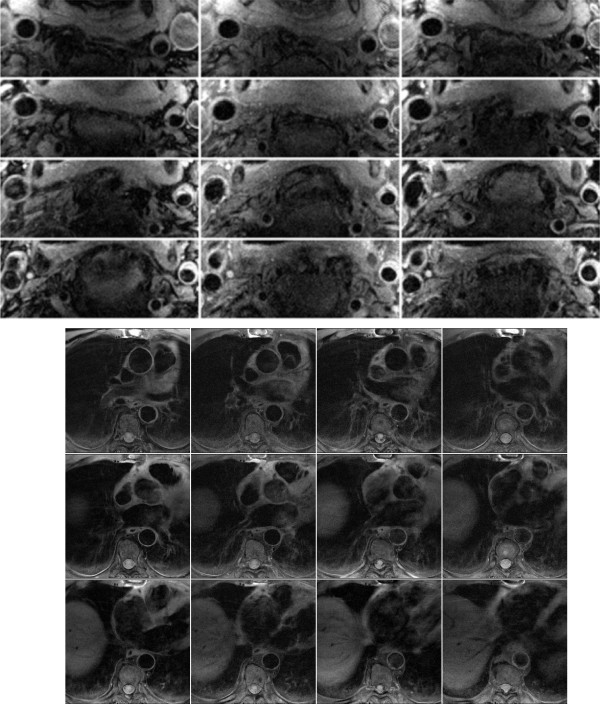
**Sample carotid images obtained from a patient (Top panels) and Sample thoracic aorta images (Bottom panels)**.

Sample images and the manual tracing of the contours are shown in Figure 1.

### Statistical Analysis

The summary statistics for the data are presented as mean ± standard deviation (SD). The mean area of the whole artery is the sum of all areas divided by the number of slices. All changes (both absolute and percentages) are presented as annualized. Statistical analysis was performed with SPSS (vs 14.0 SPSS Inc., Chicago, Illinois, USA). Differences between two groups were evaluated by the unpaired t-test for continuous variables and by the chi-square test for categorical variables. The one-sample t-test was used for the comparison of annual change to zero. For all tests, a p < 0.05 was considered statistically significant.

## Results

**Figure 2:**
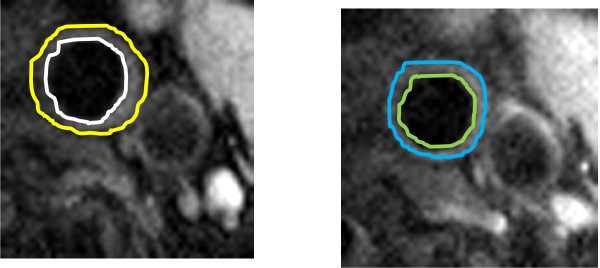
**Sample image analysis tracing of the left carotid artery showing plaque regression between the two points on a 56-year-old patient on statin therapy**.

In the carotid artery, 23 subjects and 335 slices (right 179 and left 156) were analyzed. For the thoracic aorta, 19 subjects and 141 slices were analyzed. In the abdominal aorta, 15 subjects and 106 slices were analyzed. Sample image analysis tracing of the left carotid artery showing plaque regression between the two points on a 56-year-old patient on statin therapy is shown in Figure 2.

### Demographics and Risk Factors

Table 1 provides demographic information, including the risk factor profile and lipid profile of the study population subjects among carotid aorta, thoracic aorta, and abdominal aorta. There were no significant differences in demographics' and risk factors among the subjects of carotid artery, thoracic aorta, and abdominal aorta.

### Annual Progression Rate

Tables 2, 3, and 4 demonstrate the annual progression rate of atherosclerosis and change in lumen size per year for the carotid artery, thoracic aorta, and abdominal aorta. For the carotid artery, mean total vessel area increased by 1.00 mm^2^/year (2.44%/year, p = 0.004), mean wall area increased by 1.53 mm^2^/year (8.67%/year, p < 0.001) but mean lumen area either remained unchanged or slightly decreased by -0.53 mm^2^/year (-1.23%/year, p = 0.07). For the thoracic aorta, mean total vessel area increased by 9.19 mm^2^/year (2.36%/year, p = 0.009), mean wall area increased by 9.88 mm^2^/year (10.64%/year, p < 0.001) but mean lumen area remained unchanged (-0.69 mm^2^/year (0.09%/year, p = ns)). For the abdominal aorta, mean total vessel area increased by 21.26 mm^2^/year (6.77%/year, p < 0.001), mean wall area increased by 11.23 mm^2^/year (13.24%/year, p < 0.001) and mean lumen area also increased by 10.03 mm2/year (4.97%/year, p = 0.002).

**Table 2:** Progression rate per year for Carotid artery

	Baseline ± 1SD	Δ/year absolute ± 1SD	Δ/year in % ± 1SD	p*
**Means (Data based on all matched locations)**				
Lumen [mm2]	31.18 ± 8.98	-0.53 ± 4.26	-1.23 ± 12.34	0.07
Wall [mm2]	25.00 ± 9.61	1.53 ± 9.37	8.67 ± 32.54	<0.001
Total Vessel [mm2]	56.17 ± 17.11	1.00 ± 10.72	2.44 ± 15.35	0.004
Normalized Wall Index	0.44 ± 0.06	0.02 ± 0.07	5.11 ± 16.53	<0.001

*One sample t-test vs. 0 for percentage change/year

**Table 3:** Progression rate per year for thoracic aorta

	Baseline ± 1SD	Δ/year absolute ± 1SD	Δ/year in % ± 1SD	p*
**Means (Data based on all matched locations)**				
Lumen [mm2]	359.10 ± 85.72	-0.69 ± 43.94	0.09 ± 11.98	ns
Wall [mm2]	120.07 ± 39.78	9.88 ± 20.40	10.64 ± 17.97	<0.001
Total Vessel [mm2]	479.17 ± 118.78	9.19 ± 51.72	2.36 ± 110.61	0.009
Normalized Wall Index	0.25 ± 0.04	0.02 ± 0.04	8.23 ± 15.04	<0.001

*One sample t-test vs. 0 for percentage change/year

**Table 4:** Progression rate per year for abdominal aorta

	Baseline ± 1SD	Δ/year absolute ± 1SD	Δ/year in % ± 1SD	p*
**Means (Data based on all matched locations)**				
Lumen [mm2]	259.63 ± 85.89	10.03 ± 48.49	4.97 ± 15.86	0.002
Wall [mm2]	103.48 ± 33.72	11.23 ± 15.56	13.24 ± 17.36	<0.001
Total Vessel [mm2]	399.11 ± 112.33	21.26 ± 53.20	6.77 ± 13.85	<0.001
Normalized Wall Index	0.26 ± 0.04	0.01 ± 0.04	6.45 ± 14.87	<0.001

*One sample t-test vs. 0 for percentage change/year

Briefly, the wall area significantly increased for carotid artery, thoracic aorta, and abdominal aorta. The lumen area significantly increased for abdominal aorta, but didn't change significantly for carotid arteries and thoracic aorta. This implies that different vascular locations may exhibit varying progression of atherosclerosis and remodeling as monitored by CMR.

### Demographics and Risk Factor with and without statintherapy

Previous studies suggest that use of statin shows regression of atherosclerotic lesions [27-30]. We therefore analyzed the demographic information, risk factor, and lipid profile with and without statin use.

Table 5 shows demographics and risk factors with and without statin therapy for patients with carotid artery imaging. There was no significant difference between patients with and without statin therapy with regard to other demographics and risk factors including serum lipid level. Table 6 showed demographic and risk factors for patients with and without statin therapy for the thoracic aorta imaging. Regarding gender, everyone on statin therapy was male (male gender: 100% on statin therapy vs. 54.5% not on statin therapy, p = 0.04). There were no significant differences between patients with and without statin therapy with regard to other factors including serum lipid levels. Table 7 showed demographics and risk factors with and without statin therapy for patients who underwent abdominal aorta imaging. No significant differences between those with and without statin therapy were observed in demographics and risk factors.

**Table 5:** Comparison of clinical data for patients with and without statin therapy for Carotid

Demographics and risk factors	Mean ± S.D or % Statin(-)	(Range or dosage) Statin(+)	p
Age (years)	57.0 ± 12.5 (64-27)	62.7 ± 4.9 (72-57)	ns
Male sex (%)	64.3	70.0	ns
Height (m)	1.71 ± 0.09 (1.83-1.55)	1.74 ± 0.05 (1.83-1.68)	ns
Weight (kg)	72.2 ± 14.6 (90-52)	84.7 ± 17.7 (114-62)	ns
Body Mass Index (kg/m2)	24.6 ± 3.6 (28.8-18.9)	28.0 ± 6.0 (39.2-23.4)	ns
Hypertension (%)	23.1	60.0	ns
Diabetes (%)	18.2	40.0	ns
Smoking status (%)			
Active	7.7	0.0	ns
Quit	38.5	60.0	ns
Never	53.8	40.0	ns
History of CAD (%)	30.8	20.0	ns
Hypercholesterolemia (%)	44.4	25.0	ns
Total cholesterol (mg/dl)	200.4 ± 62.5 (130-287)	176.1 ± 37.6 (128-244)	ns
LDL cholesterol (mg/dl)	127.1 ± 49.7 (65-202)	102.6 ± 29.8 (62-147)	ns
HDL cholesterol (mg/dl)	54.8 ± 16.4 (33-86)	48.0 ± 15.5 (30-77)	ns
Triglycerides (mg/dl)	106.0 ± 60.3 (46-234)	116.4 ± 63.1 (48-233)	ns

CAD: Coronary Artery Disease;

Data are mean ± S.D or % a nonpair t-test or χ^2^-test

**Table 6:** Comparison of clinical data for patients with and without statin therapy for Thoracic Aorta

Demographics and risk factors	Mean ± S.D or % Statin(-)	(Range or dosage) Statin(+)	p
Age (years)	58.5 ± 10.7 (72-35)	61.6 ± 14.0 (85-35)	ns
Male sex (%)	54.5	100.0	0.04
Height (m)	1.68 ± 0.10 (1.83-1.55)	1.77 ± 0.07 (1.89-1.68)	0.06
Weight (kg)	75.5 ± 16.0 (104-52)	82.3 ± 18.7 (123-62)	ns
Body Mass Index (kg/m2)	26.5 ± 4.4 (34.5-18.9)	26.1 ± 3.7 (34.2-22.0)	ns
Hypertension (%)	30.0	50.0	ns
Diabetes (%)	18.2	37.5	ns
Smoking status (%)			
Active	10.0	0.0	ns
Quit	30.0	50.0	ns
Never	50.0	50.0	ns
History of CAD (%)	27.3	12.5	ns
Hypercholesterolemia (%)	44.4	14.5	ns
Total cholesterol (mg/dl)	206.2 ± 57.4 (122-287)	172.1 ± 20.9 (144-205)	(0.16)
LDL cholesterol (mg/dl)	127.6 ± 49.2 (65-202)	94.3 ± 17.5 (66-114)	(0.11)
HDL cholesterol (mg/dl)	54.0 ± 17.4 (32-86)	47.9 ± 16.6 (30-78)	ns
Triglycerides (mg/dl)	136.4 ± 94.3 (46-335)	135.1 ± 82.9 (48-257)	ns

CAD: Coronary Artery Disease;

Data are mean ± S.D or % a nonpair t-test or χ^2^-test

**Table 7:** Comparison of clinical data for patients with and without statin therapy for Abdominal Aorta

Demographics and risk factors	Mean ± S.D or % Statin(-)	(Range or dosage) Statin(+)	p
Age (years)	58.6 ± 12.0 (75-35)	70.2 ± 10.0 (85-60)	0.07
Male sex (%)	55.6	66.7	ns
Height (m)	1.69 ± 0.10 (1.83-1.55)	1.72 ± 0.09 (1.78-1.55)	ns
Weight (kg)	76.3 ± 16.7(104-52)	84.3 ± 15.0 (114-74)	ns
Body Mass Index (kg/m2)	26.5 ± 4.6 (34.5-18.9)	29.4 ± 6.0 (39.2-24.4)	ns
Hypertension (%)	25.0	50.0	ns
Diabetes (%)	22.2	66.7	ns
Smoking status (%)			
Active	22.2	0.0	ns
Quit	22.2	40.0	ns
Never	55.6	60.0	ns
History of CAD (%)	33.3	16.7	ns
Hypercholesterolemia (%)	33.3	0.0	ns
Total cholesterol (mg/dl)	188.7 ± 57.9 (122-271)	157.5 ± 21.5 (128-175)	ns
LDL cholesterol (mg/dl)	111.0 ± 48.6 (65-195)	74.5 ± 14.7 (62-95)	(0.19)
HDL cholesterol (mg/dl)	55.3 ± 21.6 (32-86)	56.8 ± 16.0 (41-78)	ns
Triglycerides (mg/dl)	133.1 ± 96.6 (46-335)	184.2 ± 180.7 (48-500)	ns

CAD: Coronary Artery Disease;

Data are mean ± S.D or % a non-paired t-test or χ^2^-test

### Annual Progression Rate with and without statin therapy

Table 8 shows annual progression rates comparing subjects with and without statin therapy for patients with carotid arteries imaging. Mean lumen area of the base line was significantly higher in subjects without statin than the subjects with statin (32.28 ± 7.92 vs. 29.73 ± 10.00 p < 0.01). The progression rate of lumen area was significantly elevated in the subjects without statin therapy than the subjects with statin therapy (0.19 ± 12.91 vs. -3.08 ± 11.34 p < 0.05). There was no significant difference in the rate of change of wall area and total vessel area between the subjects with statin therapy and without statin therapy.

**Table 8:** Comparison of subjects with and without statin therapy in Carotid area

	Baseline ± 1SD			Δ/year absolute ± 1SD			Δ/year in % ± 1SD		
	Statin(-)	Statin(+)	p-value	Statin(-)	Statin(+)	p-value	Statin(-)	Statin(+)	p-value
**Means (data based on all matched locations)**									
Lumen (mm2)	32.28 ± 7.92	29.73 ± 10.00	<0.01	-0.16 ± 4.59	-1.03 ± 3.77	ns	0.19 ± 12.91	-3.08 ± 11.34	<0.05
Wall (mm2)	25.46 ± 10.01	24.39 ± 9.05	ns	1.35 ± 11.55	1.78 ± 5.33	ns	8.62 ± 38.23	8.72 ± 23.19	ns
Total vessel (mm2)	57.74 ± 16.33	54.12 ± 17.93	ns	1.19 ± 13.63	0.75 ± 4.75	ns	3.16 ± 18.61	1.50 ± 9.52	ns
Normalized wall index	0.43 ± 0.06	0.45 ± 0.06	<0.05	0.01 ± 0.08	0.02 ± 0.06	ns	4.19 ± 17.58	6.30 ± 14.98	ns

Table 9 shows the annual progression rate comparison of subjects with and without statin therapy with thoracic aorta imaging. Mean lumen area of the baseline was significantly higher in the subjects without statin therapy than the subjects with statin therapy (372.93 ± 84.22 vs. 342.91 ± 85.25 p = 0.03). There were no significant differences in the rate of change of lumen area, wall area and total vessel area between the subjects with statin and without statin therapy.

**Table 9:** Comparison of subjects with and without statin therapy in Thoracic Aortic area

	Baseline ± 1SD			Δ/year absolute ± 1SD			Δ/year in % ± 1SD		
	Statin(-)	Statin(+)	p-value	Statin(-)	Statin(+)	p-value	Statin(-)	Statin(+)	p-value
**Means (data based on all matched locations)**									
Lumen (mm2)	372.93 ± 84.22	342.91 ± 85.25	0.03	2.81 ± 49.89	-4.78 ± 35.71	ns(0.31)	1.58 ± 12.18	-1.66 ± 11.60	ns(0.11)
Wall (mm2)	117.95 ± 29.61	122.55 ± 49.22	ns	10.21 ± 22.42	9.48 ± 17.93	ns	11.14 ± 18.88	10.07 ± 16.96	ns
Total vessel (mm2)	490.89 ± 107.49	465.47 ± 130.27	ns	13.03 ± 60.62	4.70 ± 38.81	ns(0.34)	3.63 ± 11.57	0.86 ± 9.24	ns(0.12)
Normalized wall index	0.24 ± 0.03	0.26 ± 0.04	0.01	0.02 ± 0.03	0.02 ± 0.04	ns	7.13 ± 14.39	9.52 ± 15.79	ns

Table 10 shows the annual progression rates for subjects with and without statin therapy with abdominal aorta imaging. Mean lumen area, mean wall area and total vessel area of the baseline was significantly smaller in subjects without statin than the subjects with statin (lumen area: 268.11 ± 72.41 vs. 334.41 ± 89.14 p < 0.001, wall area: 86.39 ± 20.10 vs. 127.56 ± 34.48 p < 0.001, total vessel area: 354.50 ± 87.50 vs. 461.98 ± 114.17 p < 0.001). There were no significant differences in the rate of change of lumen area, wall area and total vessel area between the subjects with statin and without statin therapy.

**Table 10:** Comparison of subjects with and without statin therapy in Abdominal Aortic area

	Baseline ± 1SD			Δ/year absolute ± 1SD			Δ/year in % ± 1SD		
	Statin(-)	Statin(+)	p-value	Statin(-)	Statin(+)	p-value	Statin(-)	Statin(+)	p-value
**Means (data based on all matched locations)**									
Lumen (mm2)	268.11 ± 72.41	334.41 ± 89.14	<0.001	8.59 ± 57.32	12.06 ± 32.77	ns	5.02 ± 18.65	4.89 ± 10.97	ns(0.11)
Wall (mm2)	86.39 ± 20.10	127.56 ± 34.48	<0.001	11.32 ± 16.74	11.11 ± 13.92	ns	15.65 ± 20.23	9.85 ± 11.64	ns
Total vessel (mm2)	354.50 ± 87.50	461.98 ± 114.17	<0.001	19.91 ± 61.96	23.17 ± 38.20	ns	7.18 ± 16.31	6.18 ± 9.50	ns
Normalized wall index	0.25 ± 0.04	0.28 ± 0.05	<0.001	0.02 ± 0.04	0.01 ± 0.02	ns	8.58 ± 17.77	3.45 ± 8.73	ns(0.08)

## Discussion

This study demonstrates that in vivo CMR can quantify changes in atherosclerosis and remodeling simultaneously in multiple vascular beds, i.e., the carotid arteries, thoracic aorta, and abdominal aorta. Results indicated that different vascular beds have different rates of progression/regression under current standard of care.

Previous studies that use CMR have detected changes of atherosclerotic lumen and wall dimension in carotids. Saam et al[21] observed 74 subjects having 50-79% stenosis of carotids for 18 months. Results of that study indicated no significant change of mean total vessel area, a significant increase of 1.0 ± 2.9 mm^2 ^per year (p = 0.001) of mean wall area and decrease of 0.6 ± 2.2 mm^2 ^per year (p = 0.02) of mean lumen area. The results of the current study however showed a non significant mean lumen area decrease of 0.53 ± 4.26 mm^2 ^per year. We also observed a decrease in carotid artery lumen areas indicating progressing atherosclerosis. In terms of mean total vessel area and mean wall area, our results were comparable to those obtained by Saam et al [21]. By contrast, our subjects had a baseline normal wall index of 0.44 ± 0.06, while the subject's of Saam et al showed baseline of normal wall index of 0.63 ± 0.09 indicating that our study population was not as advanced in atherosclerotic disease stage. A lower normalized wall index has been associated with a significantly reduced rate of progression in mean wall area of carotid artery [21].

Other studies that use CMR have also detected changes of atherosclerotic lumen and wall dimensions in subjects treated with lipid-lowering therapy [27-30]. Corti et al. reported an 18% reduction in carotid wall area, a 5% increase in carotid lumen area and a 15% reduction in thoracic aorta wall area, 6% increase in thoracic aorta lumen area in 51 subjects after two years of treatment with 20 or 80 mg simvastatin. Total vessel area decreased until 12 months of treatment for thoracic aortic lesions and 18 months for carotid lesions [29,30]. This study showed the regression of atherosclerosis and changes of vessel remodeling were almost the same between carotid artery lesions and thoracic aorta lesions. The baseline of mean carotid artery wall area was 46.5 mm^2 ^(current study: 25.00 mm^2^), mean lumen area was 32.6 mm^2 ^(current study: 31.18 mm^2^) and mean thoracic wall area is 288 mm^2 ^(current study: 120.07 mm^2^), mean lumen area is 469.1 mm^2 ^(current study: 359.10 mm^2^). Corti et al also showed atherosclerotic plaque regression with statin therapy. Our study subjects had lower lipids than the subjects of the study by Corti et al. Therefore our study showed progression of atherosclerotic lesions regardless of statin therapy.

Regarding change of the mean lumen area of carotid arteries and thoracic aorta; our study showed that the subjects with statin had higher but not significantly different reduction of lumen area than the subjects without statin. On the contrary, Saam et al. showed the subjects with statin had significant reduction of mean lumen area and less regression of mean wall area than the subjects without statin in carotid artery [21]. Different severity of atherosclerosis in the multiple vascular beds may cause different changes in wall area and vessel area with or without statin.

Schoenhagen et al. [31] showed that treatment with statin was associated with constrictive remodeling in coronary artery using intravascular ultrasound. The remodeling ratio was calculated by dividing the lesion extra elastic membrane area by the reference elastic membrane area. The plaque area increased 8.9% but the remodeling ratio decreased 3.0% during 18 month follow up with atorvastatin 80 mg or pravastatin 40 mg. Statin therapy may affect vascular constrictive remodeling without regression of plaque area in carotid artery and thoracic aorta. Further studies are needed to examine this phenomenon.

Ayaori et al. [32] reported significant regression of thoracic aorta mean wall area 12 mm^2 ^but not significant change of thoracic aorta mean vessel area and significant regression of abdominal aorta mean wall area 11 mm^2 ^and significant enlargement of mean vessel area 8 mm^2 ^in 14 subjects treated with 400 mg bezafibrate for one year. The regression of atherosclerosis plaque is similar between thoracic aorta lesions and abdominal aorta lesions but the change of vessel area is different between thoracic aorta lesions and abdominal aorta lesions in subjects treated with 400 mg bezafibrate.

Yonemura et al. [28] reported significant regression of thoracic aorta mean wall area of 23 mm^2^, an enlargement of thoracic aorta vessel area of 15 mm^2 ^and an insignificant change of abdominal aorta wall area and vessel area in 19 subjects with 20 mg atorvastatin for one year. This showed the regression of atherosclerosis plaque is different between thoracic aorta lesions and abdominal aorta lesions treated with atorvastatin.

Studies have shown that plaques are more common in abdominal aorta than in the thoracic aorta [33]. In the abdominal aorta, fibrous plaques were shown by autopsy studies to be more common than in the thoracic aorta and have also been shown to increase in burden with age [34,35]. The abdominal aorta tapers geometrically and has higher pressures than the thoracic aorta. It is also stiffer with less elastin and more collagen[36]. The composition of plaque is different between thoracic aorta lesions and abdominal aorta lesions. Our results showed a greater reduction in lumen size of the abdominal aorta than that of thoracic aorta. It is also natural that the formation of aneurysm is more common in abdominal aorta than in thoracic aorta and carotid artery.

Previous studies have also shown that increased CMR plaque burden measures are related to previous major adverse cardiovascular events [37]. This would indicate that the results of the current study may potentially be useful in the evaluation of patient risk. The reproducibility of the CMR measures used in this study has been examined in the past and has been shown to be robust and reproducible [38].

### Limitations

Though this study offers insights into plaque distribution in various vascular beds, several limitations need to be noted. First and foremost, this study was a retrospective analysis performed on a limited sample size. Further prospective studies in a larger population are needed before these results can be generalized. Issues that contributed to small sample size were determined primarily by the logistics of scanning the same individual multiple times and not by image acquisition issues.

Another limitation of the current study involves analysis of mean values of plaque burden measurements over the entire artery and not within specific plaques. Specific plaques may change differently than the entire vessel as a whole and this could be the reason for some of the differences between the results obtained in this study compared to previous work. Significant differences can be observed earlier if only specific lesions are chosen for analysis as compared to the whole vessel segment. Finally, plaque characterization analysis was not performed. As a result, the effect of the presence or absence of calcification, intra plaque hemorrhage, and/or lipid rich necrotic cores on plaque progression could not be assessed. One of the reasons for the lack of plaque characterization was due to the fact that the patients in this study were at an earlier stage of atherosclerosis compared to previous studies that examined advanced disease.

Other limitations include that fact that annualized rates of progression/regression were determined using a linear model. The rates of progression and regression in individuals might vary with the age and extent of disease and need not be linear. The results of this study cannot therefore be extrapolated to determine progression/regression for a general population. Yet another limitation of this study is the fact that a regression analysis was not performed. The current analysis approach was chosen to mimic the approach by Saam et al [21,23] in order to allow for direct comparison of our data with previously published work. A multivariate regression model will provide better insights with regard to independent predictors for plaque progression. These will the basis of future work in a prospective study in a larger population.

## Conclusion

Firstly, this study demonstrates the utility of non-invasive CMR for quantifying simultaneously, changes in atherosclerotic plaques and vessel lumen size in multiple vascular beds i.e., carotid artery, thoracic aorta, and abdominal aorta. The atherosclerotic plaques progress with time (over an 18 month period) in carotid artery, thoracic aorta, and abdominal aorta. In our patient population, the lumen size increased in the abdominal aorta but did not change in carotid artery and thoracic aorta. Thus, different vascular locations exhibited varying progression/regression of atherosclerosis and remodeling. Furthermore, the fact that this study shows a lack of correlation between the change in lumen and wall areas confirms that measurement of lumen stenosis provides an incomplete picture of atherosclerosis progression and regression in any vascular bed in any subject regardless of the stage of atherosclerosis.

## List of Abbreviations

CMR: magnetic resonance Imaging; IMT: Ultrasound intima-media thickness; REX: Rapid Extended coverage turbo spin echo; PDW: proton density weighting; NWI: normalized wall index; CAD: coronary artery disease; SD: standard deviation.

## Competing interests

The authors declare that they have no competing interests.

## Authors' contributions

Conceptions and design: KH, VM, JEP, VF, ZAF. Acquisition of data: VM, AN, SHAAnalysis of data: KH, VM, ZAF. Interpretation of data: All authors. Drafting of manuscript: KH, VM. Revision of manuscript: All authors. Final approval of manuscript: All authors. Guarantors of integrity of the study: VM, ZAF
